# Supplementary material for: Mitochondrial Dysfunction in Podocytes Caused by CRIF1 Deficiency Leads to Progressive Albuminuria and Glomerular Sclerosis in Mice
Source: Int J Mol Sci. 2021 May 2;22(9):4827. doi: 10.3390/ijms22094827 (PMC8124436; doi:10.3390/ijms22094827)
Supplement: Supplementary file 1 [file ijms-22-04827-s001.zip › ijms-1201208-supplementary.pdf]

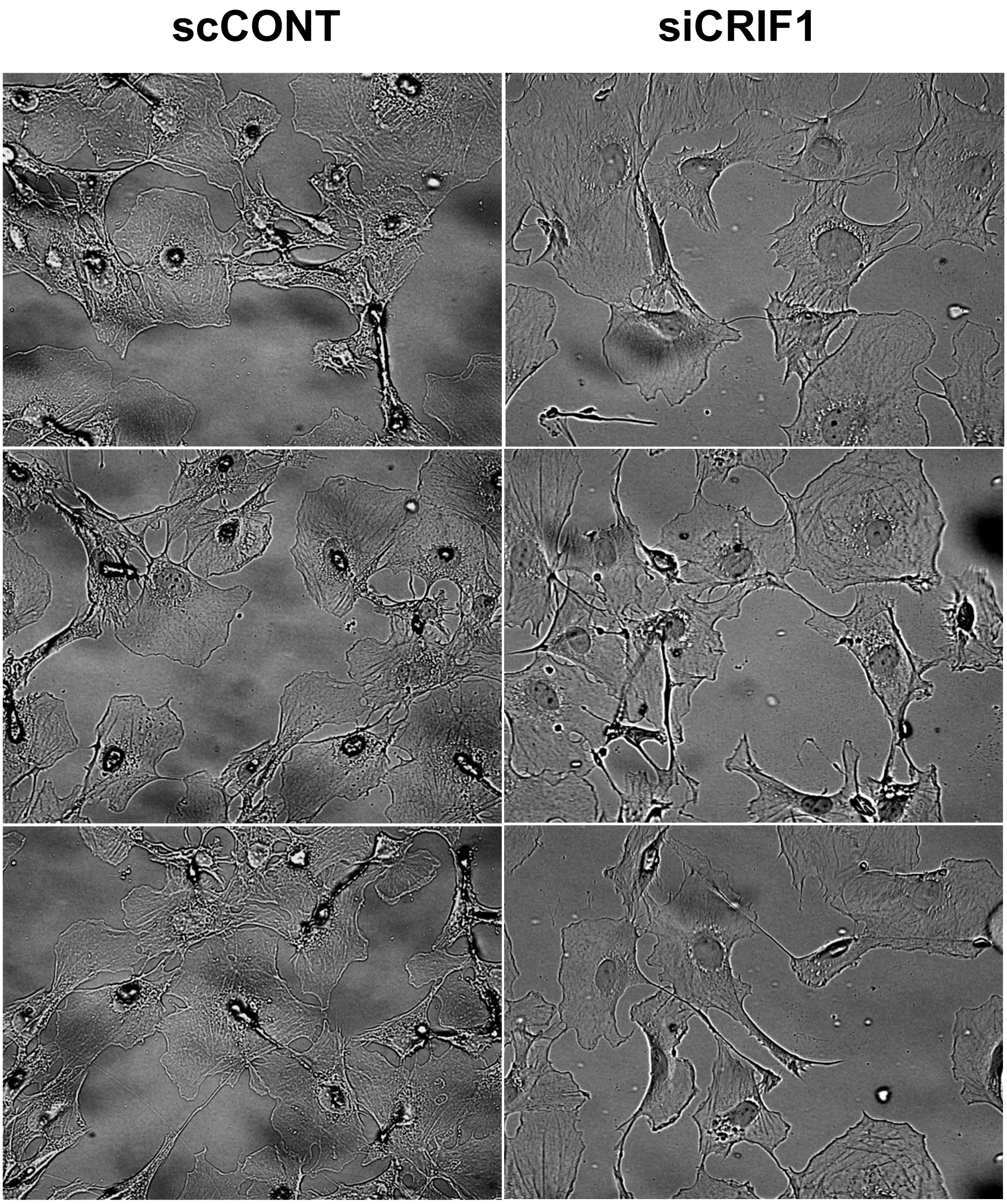

Supplementary Figure 1. Phase-contrast image of control siRNA- (scCONT) and CIRF1-specific siRNA-transfected podocytes (siCRIF1). Images of Three different area on scCONT- and siCRIF 1-podocytes were presented, respectively. No difference in the size of podocytes was observed between scCONT- and siCRIF1-podocytes.
